# Development and Validation of a UPLC-MS/MS Method to Monitor Cephapirin Excretion in Dairy Cows following Intramammary Infusion

**DOI:** 10.1371/journal.pone.0112343

**Published:** 2014-11-06

**Authors:** Partha Ray, Katharine F. Knowlton, Chao Shang, Kang Xia

**Affiliations:** 1 Department of Dairy Science, Virginia Polytechnic Institute and State University, Blacksburg, Virginia, United States of America; 2 Department of Crop and Soil Environmental Sciences, Virginia Polytechnic Institute and State University, Blacksburg, Virginia, United States of America; Auburn University, United States of America

## Abstract

Cephapirin, a cephalosporin antibiotic, is used by the majority of dairy farms in the US. Fecal and urinary excretion of cephapirin could introduce this compound into the environment when manure is land applied as fertilizer, and may cause development of bacterial resistance to antibiotics critical for human health. The environmental loading of cephapirin by the livestock industry remains un-assessed, largely due to a lack of appropriate analytical methods. Therefore, this study aimed to develop and validate a cephapirin quantification method to capture the temporal pattern of cephapirin excretion in dairy cows following intramammary infusion. The method includes an extraction with phosphate buffer and methanol, solid-phase extraction (SPE) clean-up, and quantification using ultra performance liquid chromatography-tandem mass spectrometry (UPLC-MS/MS). The LOQ values of the developed method were 4.02 µg kg^−1^ and 0.96 µg L^−1^ for feces and urine, respectively. This robust method recovered >60% and >80% cephapirin from spiked blank fecal and urine samples, respectively, with acceptable intra- and inter-day variation (<10%). Using this method, we detected trace amounts (µg kg^−1^) of cephapirin in dairy cow feces, and cephapirin in urine was detected at very high concentrations (133 to 480 µg L^−1^). Cephapirin was primarily excreted via urine and its urinary excretion was influenced by day (*P* = 0.03). Peak excretion (2.69 mg) was on day 1 following intramammary infusion and decreased sharply thereafter (0.19, 0.19, 0.08, and 0.17 mg on day 2, 3, 4, and 5, respectively) reflecting a quadratic pattern of excretion (Quadratic: *P* = 0.03). The described method for quantification of cephapirin in bovine feces and urine is sensitive, accurate, and robust and allowed to monitor the pattern of cephapirin excretion in dairy cows. This data will help develop manure segregation and treatment methods to minimize the risk of antibiotic loading to the environment from dairy farms.

## Introduction

Antibiotics are administered to livestock therapeutically to treat bacterial infections and subtherapeutically for disease prevention and growth promotion. Administered antibiotics are distributed in tissues (liver, kidney, and muscle), secreted in milk [1]–[3], and eventually excreted in feces and urine [2]. Antibiotic residues in human consumable food products can pose a threat to human health by causing toxicity or facilitating antibiotic resistance [4], [5], so maximum tolerances of antibiotics in food products of animal origin are established and monitored. Antibiotic residues in milk or meat are monitored by simple screening tests or kits designed based on microbial- or immuno-assays.

While edible products from animals treated with antibiotics have always been considered as potential human health risk factors, over the last two decades antibiotic excretion by livestock has come to be considered one of the major contributors to environmental antibiotic resistance [6]. Indirect measurements suggest that 40–90% of administered antibiotics are eliminated from animal body via feces or urine, excreted either as the parent compound or as metabolites [7]. Excreted antibiotics can persist in the environment and, even at very low concentrations, can cause emergence of antibiotic resistance in soil microorganisms, leading to dissemination of antibiotic resistance to humans and animals [8]–[11]. Therefore, reliable quantification of antibiotics excreted in feces and urine is needed assess the environmental impact of the livestock industry. The microbial- or immuno-assays used to test milk and meat may yield false positive identification in manures due to matrix interferences [12], often fail to differentiate a parent compound from its metabolite(s) [13], suffer low detection sensitivity [14]–[16], and provide only semi-quantitative results [17]. Hence, there is a need for both qualitative and quantitative analytical methods to better assess the fate and impact of antibiotics in animal products, manure, and manure-impacted environments.

Common uses of antibiotics on dairy farms are 1) dry cow therapy (prophylactic, at the end of lactation) and 2) therapeutic treatment of intramammary and other infections. In the United States, 90% of dairy farms used dry cow therapy in all cows, and cephapirin was the most frequently used compound [18]. In lactating cows, following intramammary infusion of cephapirin, 60% of the administered dose was estimated to be excreted in milk, indicating that the remaining 40% was either eliminated in urine and feces or degraded [3]. The timing of excretion following dry cow therapy is unknown, however, and may differ because these dry cows are not milked after the intramammary infusion.

In early metabolism studies, cephapirin was usually quantified using microbial inhibition immunoassay methods [1], [19]. These early methods were replaced by chromatographic methods coupled with UV-Vis detectors, pulsed amperometric detectors, mass spectrometry, or tandem mass spectrometry [20]–[23]. Most of these methods were developed to quantify cephapirin in milk and tissues, which have much higher concentrations of the antibiotic than feces and urine. Also, there are more matrix interferences from feces and urine. Therefore, established extraction, cleanup, and analytical methods for milk and tissues may not be suitable for quantification of trace amounts of cephapirin in feces and urine.

Continuous technological advancement of liquid chromatography-mass spectrometry has led to the development of new strategies to qualify and quantify antibiotics with improved selectivity and sensitivity, and therefore, tandem mass spectrometry has been preferred to other detection techniques [24]–[27]. High performance liquid chromatography is increasingly replaced with ultra high performance liquid chromatography (UPLC) to improve the resolution of chromatogram while reducing elution time and solvent consumption [28], [29].

Extraction of the analyte from sample and clean-up of sample extracts are the keys to the sensitivity of any method involving chromatography and mass spectrometry. Extractants commonly used include acetonitrile (ACN) and methanol (MeOH), separately or combined, with or without addition of buffers [20], [22], [23], [26], [28], [30]. Extraction is always followed by clean-up steps. Solid phase extraction (SPE) using cartridges is more popular than other clean-up approaches such as sample volume reduction, filtration, and dispersive SPE [20], [26], [28], [30]. The primary goal of this project was to develop an extraction, clean-up, and UPLC-MS/MS analytical method to qualify and quantify trace levels of cephapirin in bovine urine and feces. Another goal of this study was to apply the developed method to capture the temporal pattern of cephapirin excretion in dairy cows following intramammary infusion of cephapirin.

## Materials and Methods

### Chemicals and Reagents

Cephapirin standard was obtained from Sigma (Sigma, St. Louis, MO). Analytical grade monosodium phosphate, monohydrate, disodium phosphate, heptahydrate, and sodium hydroxide (1 M), and HPLC grade MeOH, ACN, and formic acid [13] were obtained from Fisher (Pittsburgh, PA).

A stock solution of cephapirin was prepared at 100 µg mL^−1^ by dissolving cephapirin in ultra pure water from the Milli-Q system (Millipore, Billerica, MA) and stored at −80°C. Intermediate stock solutions were prepared at 10 µg mL^−1^ by diluting stock solution in ultra pure water. Working solutions for instrument calibration standards and spike experiments were prepared by diluting intermediate stock solution in MeOH. A 500 mM stock phosphate buffer was prepared by dissolving 0.78 g monosodium phosphate monohydrate and 65.48 g disodium phosphate heptahydrate in 500 mL water, and the pH was adjusted to 8.5 using sodium hydroxide (1 M).

### Instrumentation

Solid phase extraction vacuum manifold and OASIS HLB (hydrophilic-lipophilic-balanced) plus short cartridge (250 mg sorbent), used for sample clean-up, were obtained from Agilent (Lexington, MA) and Waters (Milford, MA), respectively. Analyte separation and quantification were performed using Agilent 1290 UPLC coupled with Agilent 6490 Triple Quad tandem mass spectrometry (Agilent, Santa Clara, CA). Analytical (Zorbax Extend C_18_ analytical column; 4.6×50 mm, 5 µm particle size) and guard columns (Zoebax Extend C_18_ guard column; 4.6×12 mm, 5 µm particle size) were purchased from Agilent.

### Animal Experiment and Sample Collection

An experiment was conducted with dairy cows to collect samples for method development and validation and to monitor the pattern of cephapirin excretion in dairy cows following intramammary infusion. All procedures for this study were approved by the Virginia Tech Institutional Animal Care and Use Committee (IACUC protocol: 12-184-DASC). The treatment was dry cow therapy using cephapirin benzathine (TOMORROW; Boehringer Ingelheim Vetmedica, Inc., ST. Joseph, MO). Three end-of-lactation Holstein dairy cows were selected for the study, housed in a individual tie stalls (1.25×2.25 m), and offered free choice water and *ad libitum* total mixed ration throughout the study. On day 1 cows were fitted with urinary catheters to allow separate collection of feces and urine. After 24 h of acclimation to the barn and catheters, the cows were infused with 300 mg cephapirin per quarter intramammary once, per manufacturer instructions.

Blank fecal and urine samples were collected from dairy cows before cephapirin infusion and used for spike recovery experiments. Post-treatment fecal and urine samples were collected at 4, 6, and 8 h post-treatment and used to evaluate applicability of the method. To monitor the extended pattern of cephapirin excretion, daily fecal and urine samples were collected for 5 days, subsampled from excreta accumulated over each 24 h period. Fecal and urine samples from each cow were analyzed separately.

### Extraction and Clean-up

Fecal and urine samples were extracted using 50 mM phosphate buffer in MeOH and water with final MeOH concentration of 50%. Feces (1 g wet) or urine (1 mL) was weighed or pipetted into 50 mL polypropylene centrifuge tubes and 0.5 mL of phosphate buffer (500 mM; pH 8.5), 2 mL (for feces) or 1 mL (urine) of water, and 2.5 mL of MeOH were added sequentially to achieve a final concentration of 50 mM phosphate buffer and 50% MeOH(v/v). After adding the extractants, tubes were mixed (Vortex mixer) for 10 s and sonicated at 35°C for 15 min. After the sonication, the tubes were shaken on a horizontal shaker (Reciprocal Shaker; Model E6000; Eberbach Corp., Ann Arbor, MI) at a speed of 260 osc min^−1^ with horizontal stroke of 38 mm for 30 min at ambient temperature (∼20°C). After shaking, the samples were then centrifuged at 30,000×*g* for 15 min at 4°C. All supernatants were decanted into glass tubes, and diluted to 50 mL using 50 mM phosphate buffer. Tubes were inverted for 4–5 times to achieve homogeneity of sample extract and phosphate buffer.

Solid phase extraction was used to further remove matrix interference from each extract. For the SPE, OASIS HLB (hydrophilic-lipophilic-balanced) plus short cartridge (250 mg sorbent; Waters, Milford, MA) and 20 port SPE vacuum manifold (Agilent, Lexington, MA) were used. A 20 mL reservoir was mounted above each cartridge to accommodate sample extract and chemicals for conditioning, washing, and elution. Cartridges were conditioned with MeOH, water, and phosphate buffer sequentially by applying vacuum (Table 1). Sample extracts were loaded onto conditioned cartridges and vacuum was controlled to achieve a flow rate of ∼3 mL per min. Following sample loading, cartridges were washed with phosphate buffer and water sequentially (Table 1). After the washing step, vacuum was applied to draw all liquid out of the cartridges and the cartridges were allowed to dry for 4 min. Next cephapirin was eluted sequentially with 3 mL MeOH and 3 mL ACN into the same tube (Table 1).

**Table 1:** Solid phase extraction conditions.

	Step	Solvent	Volume, mL	Destination
1	Conditioning	Methanol	3	Discard
2	Conditioning	Ultra pure water	3	Discard
3	Conditioning	Phosphate buffer (50 mM, pH 8.5)	3	Discard
4	Washing^1^	Phosphate buffer (50 mM, pH 8.5)	2	Discard
5	Washing^1^	Ultra pure water	2	Discard
6	Elution^1^	Methanol	3	Collect
7	Elution^1^	Acetonitrile	3	Collect

1 Flow rate was ∼3 mL/min for washing and elution steps.

Eluted extracts were mixed using a Vortex mixer (30 s) and by inverting (4–5 times). An aliquot of 1 mL eluted extract was transferred to a 10 mL glass tube and dried under a gentle stream of nitrogen gas at 35°C using a Zipvap 20 evaporator (Glas-Col, Terre Haute, IN). Then 1 mL of MeOH:water (30∶70, v/v) with 0.1% FA was added to each tube to completely dissolve the dried cephapirin residue. The 1 mL solution in each tube was mixed (Vortex mixer) for 30 s, and filtered through 0.2 µm PVDF syringe filter (Fisher, Pittsburgh, PA) into 1.5 mL amber glass HPLC vials for the UPLC-MS/MS analysis.

### UPLC-MS/MS operating Conditions and Cephapirin Qualification and Quantification

Cephapirin was analyzed using Agilent 1290 UPLC coupled with Agilent 6490 Triple Quad tandem mass spectrometry (Agilent, Santa Clara, CA, USA). Electrospray negative ionization in multiple-reaction monitoring mode was used. Zorbax Extend C_18_ analytical column (4.6×50 mm, 5 µm particle size, Agilent, Santa Clara, CA, USA) coupled with Zorbax Extend C_18_ guard column (4.6×12 mm, 5 µm particle size, Agilent, Santa Clara, CA, USA) was used for chromatographic separation. Sampler and column compartments were kept at 8 and 40°C, respectively. The injection volume was 10 µL. A gradient elution program consisting of two mobile phases (mobile phase A: 0.1% FA in water; mobile phase B: 0.1% FA in MeOH; Table 2) was used at a flow rate of 0.5 mL min^−1^. Mass spectrometry parameters are listed in Table 3. Cephapirin in positive and spiked samples were qualified by comparing LC-MS/MS spectra of samples with those of cephapirin standards. Accepted variation in mass to charge ratio was 10%, and acceptable variation was set as 20% for the ratio of qualifier and quantifier ions. Cephapirin concentration in tested samples was quantified using the calibration curve of seven matrix-matched cephapirin standards (0.7, 1, 2, 4, 5, 10, and 20 µg L^−1^ matrix solution). Matrix-match standards were prepared using the SPE cleaned-up extracts of blank feces or urine samples.

**Table 2:** Chromatographic conditions.

	Mobile phase	
Time (min)	A^1^, %	B^2^, %
0	70	30
6	5	95
7.5	70	30
12	70	30

1 A: 0.1% formic acid in water.

2 B: 0.1% formic acid in methanol.

**Table 3:** MS/MS operating conditions.

Parameters	
Ionization mode	Electrospray negative ionization
Data collection	Multiple-reaction monitoring (MRM)
Nebulizer gas flow	16 L/min
Capillary voltage	3000 V
Fragmentation voltage	380 V
Collision energy	15 V
Ion source temperature	250°C
Precursor ion (m/z)	424
Qualifier ion (m/z)	181
Quantifer ion (m/z)	292

### Method Validation

Limit of quantification (LOQ) and limit of detection (LOD) were determined using the equations: LOQ = 10(SD/S) and LOD = 3.3(SD/S) [31], where S is the slope of a calibration curve of seven matrix-match standards and the SD is the standard deviation of responses from seven replicates of the lowest matrix-match standard. Matrix effect (%) was calculated using the equation: [{(peak area of cephapirin in matrix/peak area of cephapirin in solvent)−1}×100].

Linearity of the instrument was checked by analyzing nine cephapirin standards (1–500 µg L^−1^) prepared in MeOH:ACN (50∶50, v/v) with each concentration injected three times. A calibration curve was constructed by plotting peak areas for the standards against its concentrations. The calibration equation and correlation coefficient from the regression analysis were used to validate linearity.

Spike recovery tests were performed by spiking matrix-match standards to 1 g feces (wet weight) or 1 mL urine before extraction (pre-extraction) and in extracts (post-extraction). Three different spike concentrations were selected based on the LOQ values for cephapirin in feces and urine matrix [31]. For pre-extraction spike tests, 1 g feces or 1 mL urine was spiked with 1 mL of spike solutions (prepared in MeOH) to achieve concentrations of 2.5, 5, and 10 LOQ and was equilibrated for 2 min before extractant was added to each spiked sample. The extraction, cleanup, and analysis procedures were as described in the previous sections. For post-extraction recovery tests, feces or urine extracts were spiked at concentrations of 2.5, 5, and 10 LOQ. Spiked extracts were equilibrated for 2 min and mixed using a Vortex mixer to achieve homogenous mixing followed by clean-up and analysis using the procedures described previously.

Intra-day precision was evaluated by analyzing, at different times within one day, six replicates of cephapirin-spiked blank samples (feces or urine) at three concentrations (2.5, 5, and 10 LOQ). Inter-day precision was assessed on four different days by preparing and analyzing three replicates of cephapirin-spiked blank feces or urine at 2.5, 5, and 10 times of their respective LOQ values. Matrix effect of feces and urine was evaluated by comparing the peak area response of seven cephapirin standards dissolved in MeOH:water (30∶70, v/v, 0.1% FA) with the peak area response of those dissolved in blank fecal and urine extracts at a concentration range of 1–50 µg L^−1^.

All calibration standards were dried under N_2_ and redissolved in 1 mL MeOH:water (30∶70, v/v, 0.1% FA) using the same procedures as for the SPE cleaned-up sample extracts in order to eliminate variation due to any loss of cephapirin during the N_2_ drying process.

### Statistical Analysis

Recovery values and cephapirin concentrations in samples are reported as arithmetic means of triplicates with standard deviation calculated using Microsoft Excel. Linear regression analysis was performed in Microsoft Excel to test linearity of calibration curve. Precision was estimated as residual standard deviation using Microsoft Excel.

Excretion data were analyzed using the GLIMMIX procedure in SAS (SAS Inst. Inc., Cary, NC) with cow (n = 3) as the experimental unit. The statistical model included day as fixed effect, cow as a random variable, and pre-treatment data as a covariate. Orthogonal polynomial contrasts were used to test the linear, quadratic, and cubic effects of day.

## Results and Discussion

### Optimization of Extraction

The extraction step was optimized by testing different extractants used by others to extract cephapirin or cephalosporins from milk or biological fluid samples. Phosphate buffer at pH = 8.5 was tested to extract cephapirin from feces because previous studies have shown that high pH phosphate buffers (pH 8.5 to10) recovered cephapirin completely from milk, while a low pH phosphate buffer (pH 3.2) was inefficient in extracting cephapirin from egg [28], [30]. In our experiment, phosphate buffer (500 mM; pH 8.5) alone recovered <20% of cephapirin from feces when different sample to extractant weight to volume ratios (1∶1, 1∶5, and 1∶10) were tested. A mixture of methanol and 50 mM phosphate buffer (pH 8.5) at 50% (v/v) as extractant enhanced the recovery of cephapirin from feces (40%).

Previous research has shown that organic solvents such as acetonitrile, methanol, or their combination could recover >70% cephapirin from milk and serum [22], [23] so this approach was evaluated. Recovery was improved to >60% when a sample was first mixed at weight/volume ratio of 1∶5 for feces and volume/volume ratio of 1∶4 for urine with a methanol/50 mM phosphate buffer (pH 8.5) mixture (50%, v/v), and then sonicated at 35°C for 15 min followed by additional shaking for 30 min on a horizontal shaker at 260 osc min^−1^ with horizontal stroke of 38 mm.

Before the SPE clean-up step, 5 mL extract was diluted with phosphate buffer (50 mM) to 50 mL to bring the final concentration of MeOH in the diluted extract to below 10%, because organic solvent above this concentration was reported to elute cephalosporin antibiotics from SPE cartridges [32].

### Optimization of SPE Clean-up Step

The OASIS HLB cartridge (Waters, Milford, MA) was selected for this experiment because it is commonly used to reduce matrix effects in milk and tissue samples during sample clean-up for cephalosporin analysis [26], [30], [32]. The SPE clean-up step was optimized by testing different sequences and composition of conditioning, and elution solvents. The optimization process started with cephapirin-spiked water acidified with different volumes (50, 100, and 200 µL) of 2 M HCl with cartridge conditioning (with 2 mL ACN and 2 mL water), washing (with 3 mL water and 3 mL 3% ACN), and elution (3 mL ACN and 3 mL acetone) solvents fixed. With increasing volume of 2 M HCl, cephapirin recovery gradually decreased from 40 to 15%. It was determined that 50 µL of 2 M HCl was optimum for acidification. After determining the optimum acidification step for cephapirin spiked-water, different elution solvents were tested. Cephapirin recovery was <50% when cephapirin was eluted from the HLB cartridge using ACN and acetone mixed together or sequentially in different proportions (ACN:acetone, 50∶50 or 80∶20, v/v; 3 or 4 mL ACN followed by 3 or 1 mL acetone). Maximum recovery of >60% was later achieved when non-acidified cephapirin spiked-water was loaded onto HLB cartridge pre-conditioned with MeOH, water, and phosphate buffer (Table 1), followed by washing the cephapirin loaded cartridge with phosphate buffer (Table 1) and water, and sequentially eluting cephapirin off the cartridge using 3 mL MeOH and 3 mL ACN (Table 1). This optimized SPE clean-up approach was applied to clean up of cephapirin-spiked blank fecal and urine samples and resulted in recoveries of >60% and >80%, respectively (Table 4).

**Table 4:** Method validation data.

	Feces	Urine
LOQ^1^ (µg kg^−1^ or µg L^−1^)	4.02	0.96
LOD^1^ (µg kg^−1^ or µg L^−1^)	1.33	0.32
Pre-extraction spike recovery 2 (%)		
Spike level		
2.5×LOQ	73±4.7	81±3.5
5×LOQ	69±1.9	82±1.3
10×LOQ	64±2.5	84±2.7
Post-extraction spike recovery 2 (%)		
Spike level		
2.5×LOQ	96±2.8	90±5.9
5×LOQ	95±4.8	103±1.2
10×LOQ	100±0.3	94±4.6
Precision (%RSD)		
Intra-day^3^ (n = 18)	7.99	3.07
Inter-day^4^ (n = 36)	8.18	9.59

1 LOQ and LOD: **µ**g kg^−1^ wet feces or **µ**g L^−1^ urine.

2 Recoveries are given as mean±standard deviation (*n* = 3).

3 Intra-day variation was calculated using six replicates of three spike concentrations.

4 Inter-day variation was calculated using three replicates of three spike concentrations for four days.

### Method Validation and Application

The optimized extraction and clean-up steps were validated for quantification of cephapirin in cephapirin-spiked blank bovine feces and urine and those from animals administrated with cephapirin.

**Figure 1:**
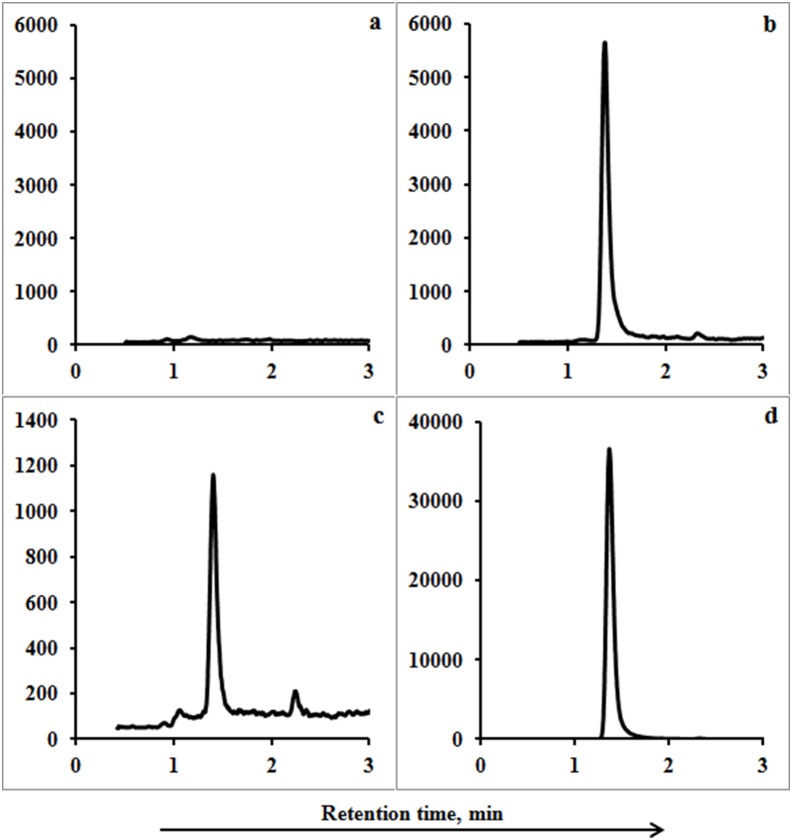
UPLC-MS/MS MRM chromatograms for cephapirin (a) in a blank fecal sample (feces from a dairy cow before treated with cephapirin), (b) in blank feces spiked with cephapirin at 10LOQ, (c) in a fecal sample collected from a dairy cow 4 h after cephapirin was administered, (d) a cephapirin standard dissolved in MeOH:water (30∶70, v/v, 0.1% formic acid) (20 µg L^−1^).

**Figure 2:**
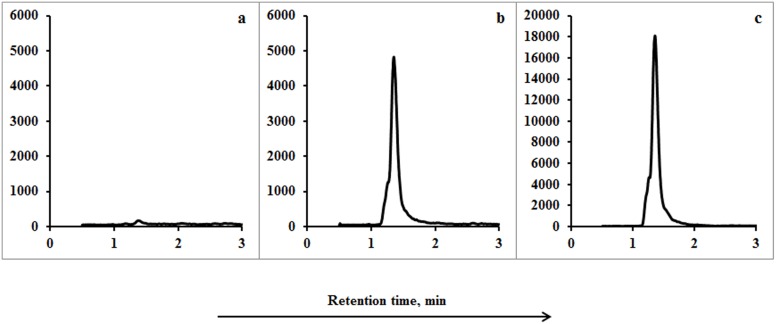
UPLC-MS/MS MRM chromatograms for cephapirin (a) in a blank urine sample (urine from a dairy cow not treated with cephapirin), (b) in blank urine spiked with cephapirin at 10LOQ, (c) in a urine sample collected from a dairy cow 4 h after cephapirin was administrated.

Figure 1 and 2 show the chromatograms of blank feces and urine from a dairy cow not treated with cephapirin, cephapirin-spiked (at 10LOQ) blank feces and urine, feces and urine collected from a dairy cow 4 h after it was treated with cephapirin, 50 µg L^−1^ cephapirin standard, and 20 µg L^−1^ cephapirin drug dissolved in solvent (this last being the form of drug administered to the cow).

**Figure 3:**
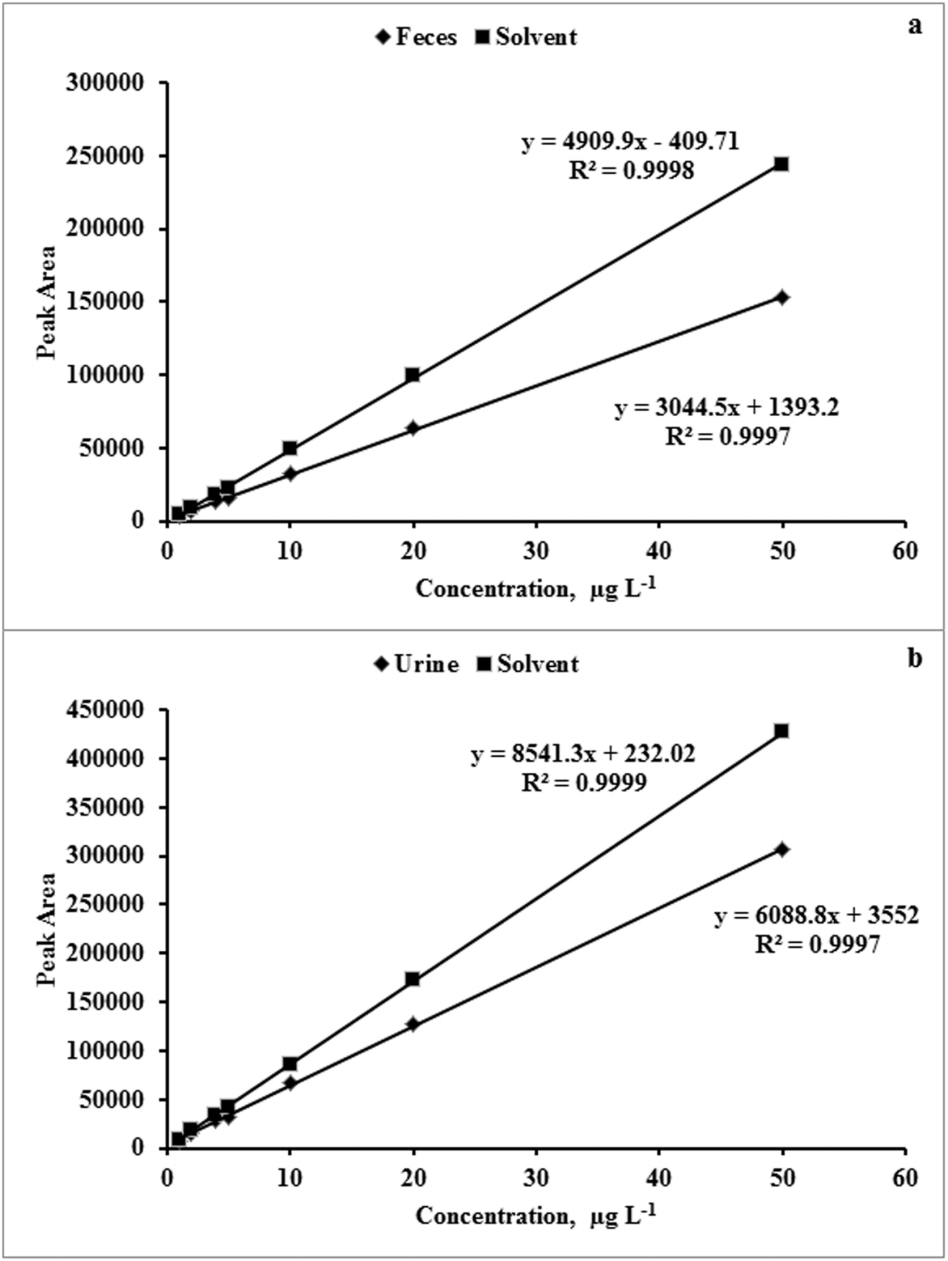
Calibration curves of standards prepared (a) in blank fecal matrix and solvent [MeOH:water (30∶70, v/v, 0.1% formic acid)]; (b) in blank urine matrix and solvent.

As shown in Figure 3, both feces and urine had matrix effects, with larger slope values for calibration curves prepared from calibration standards dissolved in LC mobile phase solvent as compared to that for cephapirin standards dissolved in fecal or urine matrix. The matrix effects for feces and urine were −29 and −20%, respectively (Table S1). Negative matrix effect indicated a suppression of response. Integration of peak area accounted for double peak and double peak area was used to plot all calibration curves or spiked-blank fecal samples. Calibration standards were prepared fresh on the day of analysis (although the standard prepared in fecal matrix at 1 µg L^−1^ was stable for one month at −20°C; data not shown). The limit of quantification for cephapirin in cephapirin-spiked blank bovine feces and urine was 4.02 µg kg^−1^ (wet weight), and 0.96 µg L^−1^, respectively.

**Figure 4:**
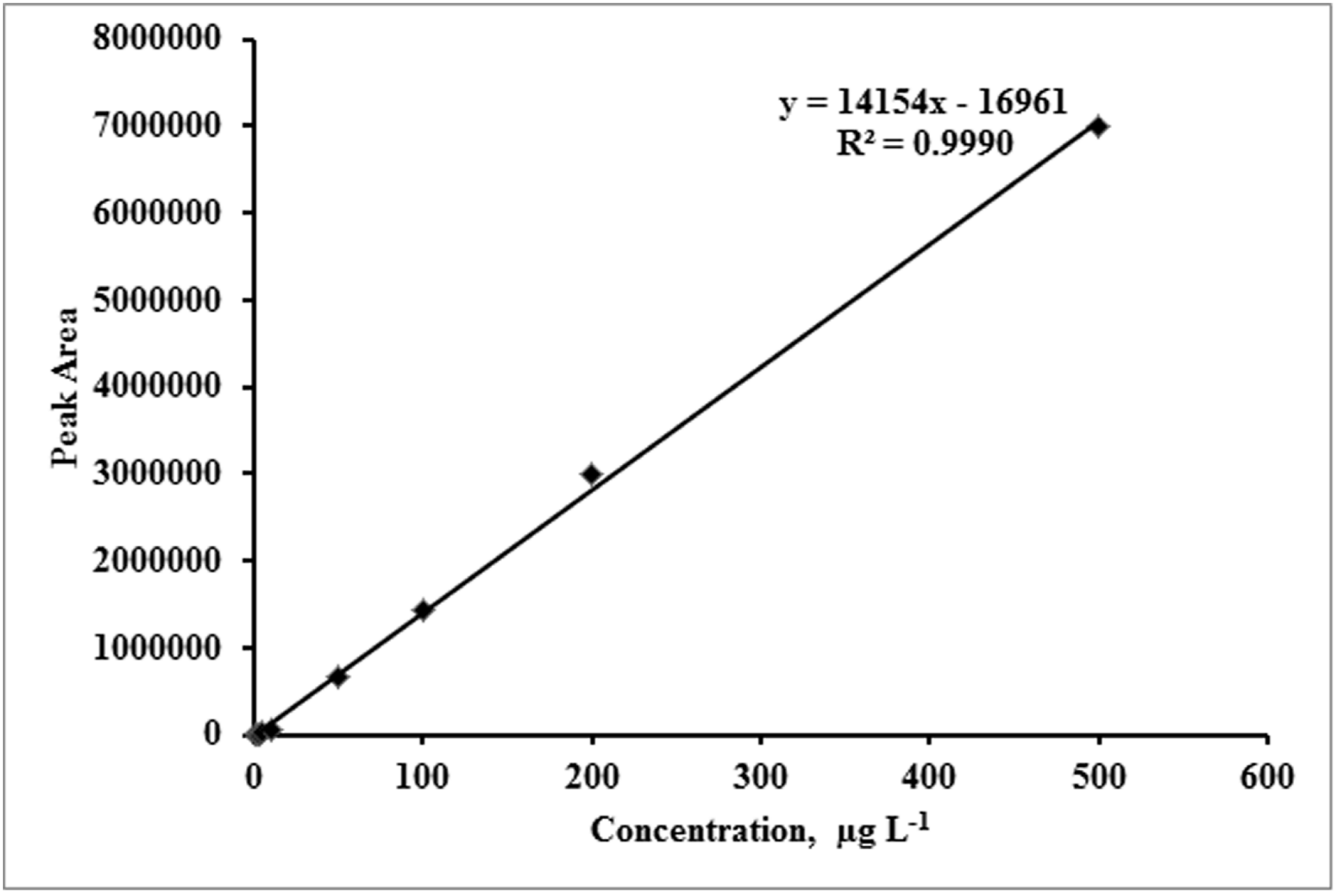
Linearity of standard curve for cephapirin standards dissolved in MeOH:water (30∶70, v/v, 0.1% formic acid) at concentrations ranging from 1 to 500 µg L^−1^.

To our knowledge, this is the first study where LOQ was determined for cephapirin in bovine feces and urine. In this experiment matrix-match calibration standards were used to reduce the effect of matrix during cephapirin quantification (Figure 3a, b). As shown in Figure 4, the instrument response was linearly correlated (r^2^ = 0.9990) with cephapirin concentration within the range of 1 to 500 µg L^−1^ (Table S2). All the standard curves used for cephapirin quantification in samples were within this range (Figure 4).

The accuracy of the method was evaluated by spike recovery tests using blank feces and urine (samples from cows before they were treated with cephapirin). The spike recovery tests were divided into two segments: pre- and post- extraction. Pre-extraction recovery tests (cephapirin spiked in blank feces or urine before extraction) were used to assess the efficiency of the entire method including extraction, clean-up, and quantification steps. The efficiency of the steps from SPE clean-up to quantification was evaluated using post-extraction recovery tests by spiking cephapirin in the extracts of blank feces or urine. Pre-extraction recovery of cephapirin in blank feces and urine samples ranged from 64 to 73% and 81 to 84%, respectively (Table 4; Table S3). Post-extraction recoveries of cephapirin were higher at 95 to 100% and 90 to103%, respectively, for feces and urine (Table 4; Table S3).

The precision of the method was evaluated in terms of repeatability (intra-day precision, % RSD) and reproducibility (inter-day precision, % RSD). The repeatability for cephapirin in spiked blank feces and urine was 7.99% and 3.07%, respectively (Table 4; Table S3), which are within the acceptable method repeatability guidelines set by FDA [33]. The reproducibility values were 8.18 and 9.59% for spiked blank feces and urine, respectively (Table 4; Table S3).

Application of the developed analytical approach was evaluated by analyzing feces and urine collected from dairy cows at 4, 6, and 8 h following intramammary infusion of cephapirin-based antibiotic. Cephapirin was detected at 2.04 and 2.12 µg kg^−1^ (wet weight) in the feces collected at 4 and 6 h post cephapirin administration, but was below the detection limit in feces collected at 8 h post cephapirin administration (Table 5; Table S4). Cephapirin concentrations were 133 and 480 µg L^−1^ in the urine samples collected at 4 and 8 h post cephapirin administration (Table 5; Table S4). Reported cephapirin concentrations in feces and urine were not normalized with their respective recoveries shown in Table 4.

**Table 5:** Quantification of cephapirin in feces and urine collected from dairy cows 4, 6, and 8 hours after they were treated with cephapirin.

	Hours after dairy cowswere treated with cephapirin	Concentration^1^(µg kg^−1^ or L^−1^)
Feces	4	2.04±0.30^2^
	6	2.12±0.09^2^
	8	ND^3^
Urine	4	133±1.35
	8	480±17.4

1 Results are given as mean±standard deviation (*n* = 3).

2 Cephapirin concentration in 4 and 6 h fecal samples are > LOD but < LOQ.

3 ND = Below LOD.

In spite of its primary use as a veterinary antibiotic, cephapirin may cause development of resistance to antibiotics critical for human health, because exposure to one antibiotic compound can cause bacteria to develop resistance to other antibiotics [34], [35]. In addition, cephapirin-induced antibiotic resistance genes in the animal gut, manure, or soil may be acquired by bacteria pathogenic to humans. The application of this method to feces and urine from antibiotic-treated cattle will aid in efforts to identify environmental practices (manure treatment, runoff control measures) to reduce loading of antibiotics to the environment. Therefore, the development of this method and improved methods to measure other antibiotics in manure has direct implications for public health.

### Temporal Pattern of Cephapirin Excretion

Cephapirin was not detected in the 24 h cumulative samples of feces collected for 5 days following intramammary infusion of cephapirin. This is because cephapirin benzathine was synthesized with the sole focus on intramammary infusion. During the synthesis of any drug that is intended for local therapy, a goal is that the drug should not reach non-target areas (in this case, the digestive tract). Therefore, local intramammary infusion ideally would not lead to significant excretion in the feces. Also, any cephapirin entering the digestive tract would be subject to degradation by intestinal bacteria. The enzyme β-lactamase, active on cephalosporins, was detected in bacteria isolated from the digestive tract of cows not exposed to antibiotics, and cephalosporins (ceftiofur and ceftriaxone) were degraded in the presence of those bacterial isolates [36]. Cephalosporin (ceftiofur) was degraded almost completely within 8 h when incubated at room temperature in the presence of bovine feces [37]. The critical role of fecal microbes in degrading cephalosporin was confirmed by low-extent of degradation (∼40%) of ceftiofur in the presence of sterile feces even after 48 h of incubation [37].

**Figure 5:**
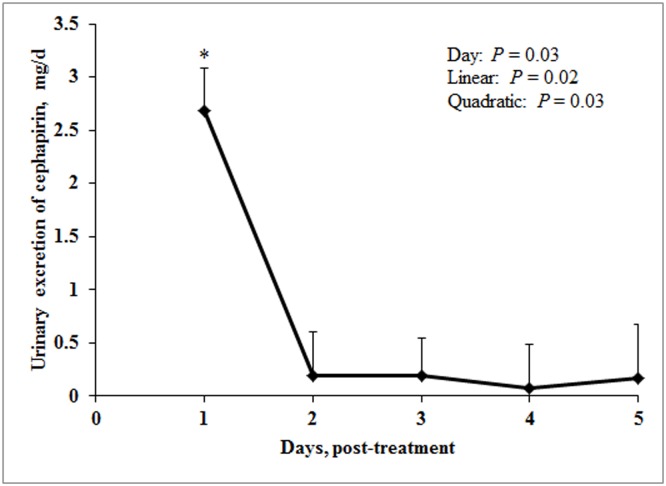
Daily urinary excretion of cephapirin (mg) in dairy cows administered with cephapirin (300 mg/quarter) via intramammary route. Data is represented as least square means with error bars as standard errors. Symbol * indicates that day 1 is significantly (*P*<0.05) different from all other days.

Urine was the primary route of cephapirin excretion in lactating cows as has been observed in other animals including humans [38]–[41]. Cephapirin excretion in urine was influenced by day (*P* = 0.03) with peak excretion (2.69 mg) on day 1 following intramammary infusion (Figure 5). After the peak on day 1, cephapirin excretion decreased sharply on day 2 (0.19 mg) and did not change for rest of the study (0.19, 0.08, and 0.17 mg on day 3, 4, and 5, respectively; Table S5) reflecting quadratic pattern of urinary cephapirin excretion (Quadratic: *P* = 0.03; Figure 5).

Drug molecules are transported from milk to blood primarily via passive diffusion [42]. Initial peak urinary excretion of cephapirin on day 1 post-treatment was likely due to faster diffusion of cephapirin along high concentration gradient from its high concentration in the milk to low concentration in the blood.

Passive diffusion of drugs across the milk-blood barrier requires wide distribution of the drug throughout the udder. This is regulated by three characteristics of the drug 1) lipid solubility, 2) rate and extent of ionization, and 3) milk protein or tissue binding [43]. Low lipid solubility of cephapirin may explain low absorption of cephapirin in this study, but does not explain the temporal variation in urinary excretion as any change in cephapirin solubility is not expected over the time. It would be expected that milk pH remained relatively stable during these experiments because cows did not have clinical mastitis and were not subject to dietary changes. Therefore, temporal variation in cephapirin excretion cannot be attributed to the change in ionization rate of cephapirin due to pH variation, so binding to tissue or milk proteins is the likely explanation. Drugs or antibiotics diffuse across the milk-blood barrier only in the unbound form. When antibiotics bind to udder tissue or milk protein, only a small proportion of the administered dose is available for absorption [44]–[46]. Peak excretion of cephapirin in urine within 24 h of intramammary infusion of cephapirin suggests saturation of binding sites immediately post-treatment due to the initial high concentration of cephapirin, leaving a major proportion of the administered dose available for absorption. Similarly, tetracycline absorption from bovine udders gradually decreased with time following infusion, an observation attributed to saturation of antibiotic binding sites [42].

After a transitory increase on day 1, the sharp decrease in cephapirin excretion on day 2 was likely due to increased binding of cephapirin to tissue protein because the extent of drug binding to protein increases with the decrease in drug concentration [47]. Also, with time, conversion of cephapirin to its major metabolite desacetyl cephapirin is likely. Within 24 h of intramammary infusion of cephapirin in lactating cows >50% of infused cephapirin was converted to desacetyl cephapirin [3], [48].

When expressed as a proportion of total dose, only 0.22% was excreted on day 1 after intramammary infusion. On following days excretion ranged from 0.01 to 0.02% of total cephapirin administered. Cumulative excretion of cephapirin for 5 days post-treatment was 0.28%. The excretion of only a very small proportion of total intramammary dose was likely due to low solubility of cephapirin leading to limited distribution throughout the udder and subsequently poor absorption [44], [49]. This low solubility is intentional to increase the duration of therapy during the dry period (∼45–60 days). Milk is mildly acidic (pH 6.4–7) and ∼99% of cephapirin is ionized at pH 6.4 [44], [50]. Therefore, only 1% would be available as nonionized (absorbable) form. Also each nonionized molecule will not necessarily be absorbed because binding of nonionized cephapirin to milk protein or tissue would limit passive diffusion of cephapirin. The large size of the cephapirin benzathine molecule is another factor that might have contributed to poor absorption of cephapirin across the milk-blood barrier. Similarly, penicillin benzathine was poorly absorbed in the bovine udder [51].

Cephapirin excretion expressed as a proportion of total dose can provide a basis of indirect comparative risk assessment of antibiotic loading to the environment, by comparison with excretion of other cephalosporins or other classes of antibiotics following systemic administration in animals and human. When administered via intravenous (IV) and intramuscular (IM) routes at 950 mg/subject in humans, the proportion of administered cephapirin excreted in urine was 48% after 6 h of IV and 43% after 24 h of IM [39]. A similar extent of cephapirin excretion was observed in humans receiving 1000 mg of cephapirin by the IV route [38]. Urinary excretion of cephapirin varied in small animals with the route of administration. In mice, 20% of the total cephapirin dose was excreted in urine within 24 h of subcutaneous administration (30 mg/kg), but the rate and extent of excretion (32% of total dose within 8 h of dosing) was higher in dogs administered with cephapirin at 30 mg/kg by the IV route [38]. Following an oral dose of cephradine, another cephalosporin, 84 and 90% of the total dose was excreted in the urine of mice and rats [41]. In dogs, within 7 h of an oral dose (50 mg/kg) of cephradine, >50% of the administered dose was excreted in feces and urine. In pigs, fecal and urinary excretion of ceftiofur ranged from 81 to 95% of total administered when IM dose varied between 3.08 and 6.76 mg/kg body weight [52]. Ceftiofur was almost completely (92.5% in urine and 6.5% in feces) excreted by sheep administered with 5 IM doses at 2.2 mg/kg body weight [53]. These cephalosporin formulations are designed to be absorbed for distribution to target tissues. In contrast, the cephapirin formulation used in this study is designed to be locally active to increase the duration of therapy during the dry period (∼45 to 60 days). Therefore, there is less chance that a higher proportion of intramammary dose would be excreted.

Even though only a small proportion of intramammary cephapirin dose was excreted in urine, the concentration range of urinary cephapirin (5.94 to 240 µg L^−1^) in this study indicated the potential of excreted cephapirin residue in exerting selection pressure on environmental microbial communities. Cephapirin is primarily used as dry cow therapy in dairy cows, and on most dairy farms in the US cows are housed on pasture or drylots during the dry period [54]. Therefore, cephapirin excreted in urine will enter the soil and its concentration in soil will depend on the distribution and transportation of cephapirin in the soil. Dose-response relationships are not established to know what concentration of which antibiotic will cause antibiotic resistance development in various bacterial species under specific environmental condition. Because of these uncertainties, all that can be discussed is the potential of excreted cephapirin concentration with respect to minimum inhibitory concentration (MIC) of cephapirin estimated in laboratory conditions.

Reported MIC of cephapirin for several bacterial species (e.g. *Staphylococcus aureus, Streptococcus uberis, Streptococcus dysgalactiae, Escherichia coli, Pseudomonas aeruginosa, Proteus mirabilis*) ranged from 30 to 64,000 µg L^−1^ when inoculum size varied from 10^4^ to 10^7^ cells mL^−1^ [55]–[60]. Our discussion will consider reported MIC values of cephapirin, but it should be kept in mind that most of these MIC values were estimated by *in vitro* susceptibility tests using nutrient rich pure growth media with fixed initial inoculum size, and MIC values vary with several factors including inoculum size and the substrates present in the media [55], [58], [61]. Since there are no established MIC values of cephapirin for soil matrices, we will discuss the potential of excreted urinary cephapirin in antibiotic resistance development considering two scenarios: cephapirin concentration 1) higher than MIC and 2) lower than MIC. The range of urinary cephapirin concentration in this study (5.94 to 240 µg L^−1^) overlaps with reported MIC values (30 to 64,000 µg L^−1^) and exceeds the MIC values of cephapirin for some bacterial species. If the soil bacterial community is exposed to a concentration of cephapirin that is higher than MIC, resistant species of bacteria will grow due to selection pressure and contribute to the environmental antibiotic resistome. The most likely scenario is that the concentration of cephapirin in soil will be much lower than the urinary concentration we observed in this study due to degradation, distribution in larger area, and adsorption to soil particles. Antibiotics are detected in the environmental samples at parts per trillion (ppt) or parts per billion (ppb) levels [27]. If cephapirin concentration in soil is far below MIC, it can still contribute to antibiotic resistance development in soil bacterial community [62], [63]. Antibiotic concentrations ranging from ¼ to 1/230 of MIC for susceptible strains exerted selection pressure on certain bacterial communities leading to increased antibiotic resistance, and this increase was attributed not only to enrichment and maintenance of pre-existing resistance but also to the *de novo* selection of new resistant traits [64].

Although intramammary infusion of cephapirin is used on >90% of dairy farms in the US, the contribution of this management practice to the environmental pool of antibiotic residue is likely lower than when the same drug is used therapeutically. If manure treatment methods are developed that are effective in degrading antibiotics, manure from cows on day 1 following dry cow therapy would be the priority for segregation and treatment. However, manure from days 2 through 5 might also need to be treated or stored for longer duration to ensure complete degradation of cephapirin, because antibiotics even at very low sub-MIC concentrations can exert selection pressure. The results of this study will help to develop efficient management strategies to reduce the development of antibiotic resistance in the environment.

## Conclusions

A method was developed and validated for qualification and quantification of cephapirin in bovine feces and urine, including extraction and clean-up, coupled with UPLC-MS/MS. This method is appropriate both qualitatively and quantitatively for detection of cephapirin in feces and urine with very low LOQ. This method can be applied to qualify and quantify cephapirin in bovine feces and urine with high accuracy. It allows measurement of trace amounts of cephapirin typical of those present in feces and urine from treated cattle, and thus will help assess environmental loading of antibiotics from the livestock industry. Urinary excretion of cephapirin followed a quadratic pattern with peak excretion on day 1 post-treatment followed by a sharp decrease on day 2. Excretion of only a minor proportion of total cephapirin dose indicates that the environmental loading of cephapirin due to intramammary cephapirin use in dairy cows is less compared to therapeutic use of other antibiotics in human and animals.

## Supporting Information

Table S1 **Effect of feces or urine matrix on cephapirin quantification.**
(PDF)

Table S2 **Linear correlation between cephapirin concentration and instrument response.**
(PDF)

Table S3 **Recovery of cephapirin spiked in feces or urine or in their extracts.**
(PDF)

Table S4 **Cephapirin concentration in feces and urine collected from dairy cows treated with cephapirin.**
(PDF)

Table S5 **Daily urinary excretion of cephapirin in dairy cows following intramammary infusion of cephapirin.**
(PDF)
