# Supplementary material for: Development and Validation of a UPLC-MS/MS Method to Monitor Cephapirin Excretion in Dairy Cows following Intramammary Infusion
Source: PLoS One. 2014 Nov 6;9(11):e112343. doi: 10.1371/journal.pone.0112343 (PMC4223036; doi:10.1371/journal.pone.0112343)
Supplement: Table S1 — Effect of feces or urine matrix on cephapirin quantification. (PDF) [file pone.0112343.s001.pdf]

Table S1: Effect of feces or urine matrix on cephapirin quantification

Concentration (ng/mL)	Fecal matrix	Solvent	Urine matrix	Solvent
	Peak area			
1	3808	4773	7757	8128
2	7146	9106	14943	19512
4	13182	18415	28018	33401
5	16121	22728	32770	42098
10	33147	49573	67280	85138
20	63442	100016	127544	172063
50	152998	244228	306718	427088
