# Supplementary material for: Development and Validation of a UPLC-MS/MS Method to Monitor Cephapirin Excretion in Dairy Cows following Intramammary Infusion
Source: PLoS One. 2014 Nov 6;9(11):e112343. doi: 10.1371/journal.pone.0112343 (PMC4223036; doi:10.1371/journal.pone.0112343)
Supplement: Table S2 — Linear correlation between cephapirin concentration and instrument response. (PDF) [file pone.0112343.s002.pdf]

Table S2: Linear correlation between cephapirin concentration and instrument response

Concentration (ng/mL)	Peak area
1	4773
2	9106
4	18415
5	22728
10	49573
50	670010
100	1439519
200	2990213
500	6985360
