# Supplementary material for: Development and Validation of a UPLC-MS/MS Method to Monitor Cephapirin Excretion in Dairy Cows following Intramammary Infusion
Source: PLoS One. 2014 Nov 6;9(11):e112343. doi: 10.1371/journal.pone.0112343 (PMC4223036; doi:10.1371/journal.pone.0112343)
Supplement: Table S3 — Recovery of cephapirin spiked in feces or urine or in their extracts. (PDF) [file pone.0112343.s003.pdf]

Table S3: Recovery of cephapirin spiked in feces or urine or in their extracts

	Feces	Urine
Pre-extraction spike recovery (%)		
Spike level		
	67.55	83.69
2.5×LOQ	76.75	82.16
	74.11	77.02
	70.05	83.28
5×LOQ	71.07	81.15
	67.37	80.93
	66.46	83.71
10×LOQ	62.07	81.02
	62.26	86.42
Post-extraction spike recovery (%)		
Spike level		
	98.74	83.63
2.5×LOQ	93.87	95.40
	94.01	89.81
	95.51	103.9
5×LOQ	98.93	102.0
	89.44	101.7
	99.80	98.78
10×LOQ	99.91	89.73
	99.31	92.91
