# Supplementary material for: Development and Validation of a UPLC-MS/MS Method to Monitor Cephapirin Excretion in Dairy Cows following Intramammary Infusion
Source: PLoS One. 2014 Nov 6;9(11):e112343. doi: 10.1371/journal.pone.0112343 (PMC4223036; doi:10.1371/journal.pone.0112343)
Supplement: Table S4 — Cephapirin concentration in feces and urine collected from dairy cows treated with cephapirin. (PDF) [file pone.0112343.s004.pdf]

Table S4: Cephapirin concentration in feces and urine collected from dairy cows treated with cephapirin

	Post-treatment hours, h	Concentration, µg kg^{-1} or µg L^{-1}
Feces	4	2.29
		1.71
		2.13
	6	2.03
		2.19
		2.15
	8	ND
		ND
		ND
Urine	4	131.8
		134.3
		133.9
	8	462.5
		497.3
		481.6
