# Supplementary material for: Development and Validation of a UPLC-MS/MS Method to Monitor Cephapirin Excretion in Dairy Cows following Intramammary Infusion
Source: PLoS One. 2014 Nov 6;9(11):e112343. doi: 10.1371/journal.pone.0112343 (PMC4223036; doi:10.1371/journal.pone.0112343)
Supplement: Table S5 — Daily urinary excretion of cephapirin in dairy cows following intramammary infusion of cephapirin. (PDF) [file pone.0112343.s005.pdf]

Table S5: Daily urinary excretion of cephapirin in dairy cows following intramammary infusion of cephapirin[1]

Days, post-treatment	Daily cephapirin excretion in urine (mg)[2]	Standard error
1	2.69	0.4
2	0.19	0.41
3	0.19	0.36
4	0.08	0.41
5	0.17	0.51

[1]Cows were administered with 300 mg cephapirin per quarter via intramammary route.

[2]Data is presented as least square means (n=3).
